# Supplementary material for: From Chaos to Coherence: Effects of High-Order Synaptic Correlations on Neural Dynamics
Source: ArXiv. 2025 Sep 3:arXiv:2504.00300v2. Preprint. [Version 2] (PMC12425029)
Supplement: Supplement 1 [file NIHPP2504.00300v2-supplement-1.pdf]

I. SUPPLEMENTARY MATERIALS

A. Matrix generation

To generate α-order cyclic correlations, we start with an $N \times N$ random matrix, where the entries are iid and $\sim \mathcal{N}(0, g^2/N)$. Next, we methodically change the sign of the corresponding entries such that the sum of all cycles of length α that end up in that specific entry would be positive with a probability P. This probability determines the strength of the correlations ρ, see Fig. S1. We do the same for negative correlations, but demand that the sum be negative. The step-by-step description is given in Algorithm 1. Note that $w_{l,1:k}$ is a $1 \times k$ vector,

Algorithm 1: Generating high-order cyclic correlations

input : $N \times N$ random matrix with $w_{ij} \sim \mathcal{N}(0, g^2/N)$
output: $N \times N$ matrix with $\mathrm{Tr}\, W^\alpha/N = \rho > 0$

Initialize an $N \times N$ random matrix, where the entries are iid with $w_{ij} \sim \mathcal{N}(0, g^2/N)$;
for $n = \alpha - 1 : N$ **do**
  $\tilde{w} = (w_{n+1,1:n} w^{\alpha-2}_{1:n,1:n}) \odot w_{n+1,1:n}$;
  for $c = 1 : n$ **do**
    if $\tilde{w}_{c,n+1} < 0$ **then**
      $w_{c,n+1} \to -w_{c,n+1}$ by a probability P;
    end
  end
end

and $\odot$ is the element-wise product.

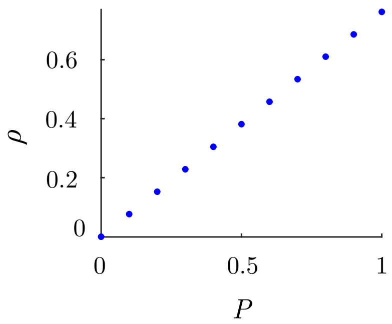

FIG. S1: Numerical value of ρ as a function of the probability P, averaged over 30 realizations.

B. Numerical details of phase diagram

The probability of observing chaotic, oscillatory, or fixed point behavior was computed by averaging the results obtained using 300 different network realizations with $N = 1600$. To determine a fixed-point solution we

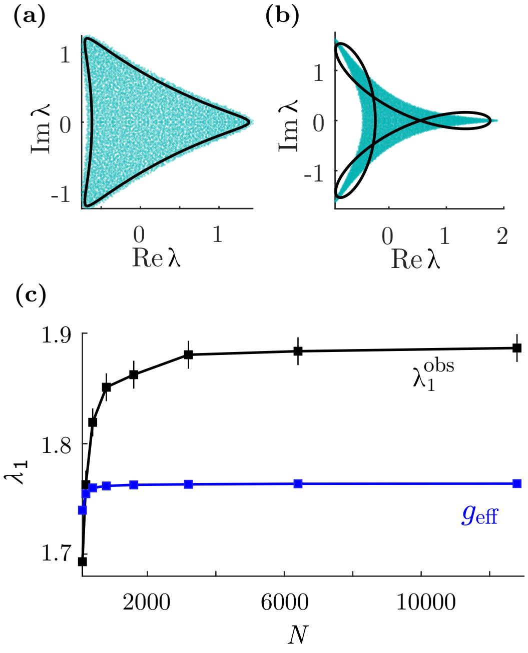

FIG. S2: (a) Eigenvalue distribution (cyan dots) shown for $\rho \approx 0.38$ with $N = 6400$. The black curve shows the solution of the equation defining the support of the eigenvalue distribution $z(\phi) = e^{i\phi} + \rho e^{-2i\phi}$. (b) The same as (a) but with $\rho \approx 0.76$. (c) The observed values of the real part of the rightmost eigenvalue, λ_1^{obs} (black) and g_{eff} (blue) as a function of N, with $g = 1$ and $\rho = 0.76$, averaged over 30 different realizations.

computed the absolute value of the numerically determined derivative of the individual trajectory, and tested if this value was smaller than 10^{-4} in the final 20% of the total runtime of the dynamics. If this test indicated that a fixed point was not reached, we estimate the Lyapunov exponents with a cutoff of 2×10^{-3} to separate chaotic from oscillatory solutions. Colors were shaded based on their corresponding probabilities with red, green, and blue, for chaos, oscillations, and fixed points respectively.

C. Probabilities of observing chaotic dynamics

The probability of observing chaotic behavior as a function of g_{eff} for various network sizes, N, is shown in Fig. S3. We classified dynamics with Lyapunov exponents exceeding 2×10^{-3} as chaotic. We used different numbers of realizations, N_R: 300 for $N \leq 2400$, 200 for $N = 3200$, 100 for $N = 4800$ and 6400, and 50 for $N = 12800$. Each curve in Fig. S3 was fitted with $P(g_{\mathrm{eff}}) = \frac{1}{2}\left[1 + \tanh\left(b\left(g_{\mathrm{eff}} - c\right)\right)\right]$. From these fits, we extracted the value of g_{eff} corresponding to $P = 0.5$.

The extracted values of g_{eff} as a function of $1/\sqrt{N}$ are plotted in Fig. S4. Each set of data points was fit to a linear model with $g_{\mathrm{eff}} = a + bN^{-1/2}$. Figure S4(a)-S4(b) shows the data corresponding to $\rho = 0$ and $\rho \approx 0.23$

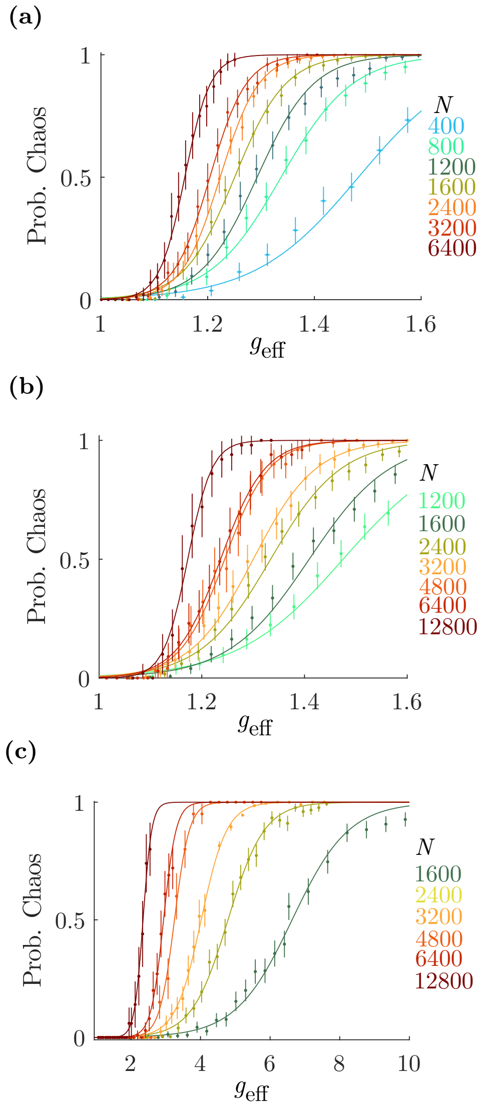

FIG. S3: Probability of observing chaotic behavior as a function of g_{eff} for different values of N with (a) $\rho \approx 0$, (b) $\rho \approx 0.23$, and (c) $\rho \approx 0.76$. Error bars depict 2σ errors, solid lines show the fit $P(g_{\text{eff}}) = \frac{1}{2}\left[1 + \tanh\big(b(g_{\text{eff}} - c)\big)\right]$.

respectively. The fitted curves in both cases show excellent agreement with a linear model ($R^2 \approx 0.99$) with an interception point slightly above $g_{\text{eff}} = 1$. Figure S4(c) depicts the data corresponding to $\rho \approx 0.76$. A linear fit fails to describe the data, so we fit the data to a model of the form $g_{\text{eff}} = a + bN^{-c/2}$. The extrapolated interception point is far from 1, showing a significant qualitative change compared with $\rho \approx 0$ and $\rho \approx 0.23$. These results support our hypothesis that chaotic behavior is suppressed at the onset of instability of the origin when correlations are strong.

In theory, as $N \to \infty$ and $P \to 1$, the expected intercept in Fig. S4(a) is $g_{\text{eff}} \to 1$. Thus, finite-size effects and the choice of $P = 0.5$ as our reference probability point may cause small deviations. The value $P = 0.5$ was chosen to avoid any numerical issues that may arise when P approaches 1. Additionally, in Fig. S3(c), the probabilities of observing chaotic behavior provide a slight overestimate of the corresponding probabilities in large networks. Independent runs reveal that even apparently highly chaotic dynamics can suddenly converge to a limit cycle or fixed point. This transient behavior becomes increasingly difficult to detect as N grows. Consequently, the values of g_{eff} in this panel should be treated as a lower bound for the true values, making chaotic behavior at large N even less probable than reported here.

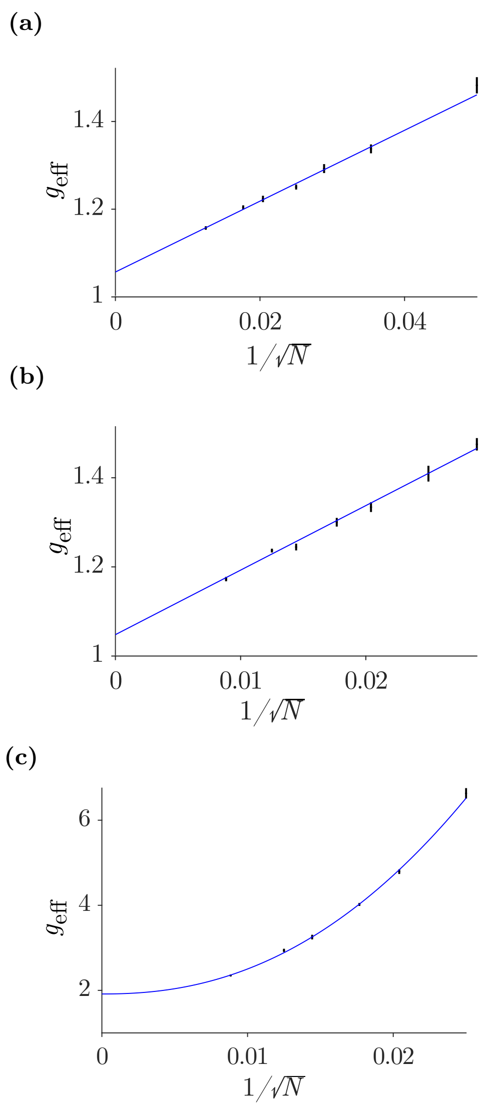

FIG. S4: Black points show g_{eff} as a function of $1/\sqrt{N}$ for (a) $\rho \approx 0$, (b) $\rho \approx 0.23$, and (c) $\rho \approx 0.76$ with 2σ error bars. The blue solid lines depict a linear fit $y = a + bx$ in (a) and (b), and a power-law fit $y = a + b\,x^c$ in (c). Fit parameters: (a) $a = 1.06 \pm 0.01$, $b = 8.1 \pm 0.3$; (b) $a = 1.05 \pm 0.01$, $b = 14.5 \pm 0.7$; (c) $a = 1.9 \pm 0.7,\ b = (1.83 \pm 1.77) \times 10^4,\ c = 2.2 \pm 0.3$.

D. Error estimation and fitting process

We first fit each set of data points in Fig. S3 to a sigmoid function of the form $P(g_{\text{eff}}) = \frac{1}{2}\big[1 + \tanh\big(b(g_{\text{eff}} -$

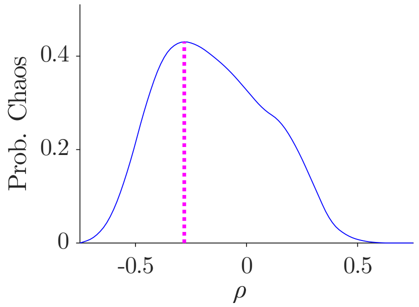

FIG. S5: Probability of observing chaotic behavior as a function of ρ for $g_{\rm eff} = 1.2$. The magenta dotted line depicts the value of the maximum probability at $\rho \approx -0.28$.

$c))\big]$. For each chosen value of $g_{\rm eff}$, there is a true value of the rightmost eigenvalue, λ. Thus, henceforth, we refer to $g_{\rm eff}$ as the mean real part of the leading eigenvalue Re λ, $\bar{\lambda}$, with a standard error $\sigma_{\bar{\lambda}} = s/\sqrt{N_R}$ from N_R realizations (with sample standard deviation s). The probability of observing chaotic dynamics, P, has a binomial standard error $\sigma_P = \sqrt{P(1-P)/N_R}$. The parameters b and c are obtained by weighted nonlinear least squares with inverse-variance weighting. To account for uncertainty in both axes, we use an effective variance $\sigma^2_{\rm eff} = \sigma_p^2 + \big[f'(g_{\rm eff})\,\sigma_{\bar{\lambda}}\big]^2$, with $f'(g_{\rm eff}) = \frac{b_0}{2}\,{\rm sech}^2\big(b_0(g_{\rm eff} - c_0)\big)$, and weight each point with $1/\sigma^2_{\rm eff}$. In practice, we implement this in two steps: A first fit using only $1/\sigma_P^2$ to obtain provisional (b_0, c_0), followed by computing $f'(\bar{\lambda})$ at (b_0, c_0) to obtain $\sigma^2_{\rm eff}$. We then refit the data with $1/\sigma^2_{\rm eff}$. The fit yields b, c and the corresponding covariance matrix Cov(b, c). We note that the horizontal error, $\sigma_{\bar{\lambda}}$ is between one to two orders of magnitude smaller than the vertical error in the probability of observing chaotic behavior, thus, practically negligible in the fitting process.

Once b and c are estimated, we invert the sigmoid at a target probability P_0, $\bar{\lambda}_{P_0} = c + \frac{1}{b}\,{\rm arctanh}(2P_0 - 1)$, and propagate uncertainties via $\sigma^2_{\bar{\lambda}_{P_0}} = \left(\frac{\partial \bar{\lambda}_{P_0}}{\partial b}\right)^2 \sigma_b^2 + \left(\frac{\partial \bar{\lambda}_{P_0}}{\partial c}\right)^2 \sigma_c^2 + 2\,\frac{\partial \bar{\lambda}_{P_0}}{\partial b}\,\frac{\partial \bar{\lambda}_{P_0}}{\partial c}\,{\rm Cov}(b, c)$. Finally, we fit $\bar{\lambda}_{P_0}$ *vs.* $1/\sqrt{N}$ to a linear model $y = a + b\,x$, or a power law model $y = a + b\,x^c$ using weights $1/\sigma^2_{\bar{\lambda}_{P_0}}$, and report the fitted parameters with their $2\,\sigma$ uncertainties, Fig. S4. In this figure we show the results for $P_0 = 0.5$, where $\bar{\lambda}_{P_0}$ was replaced with $g_{\rm eff}$.

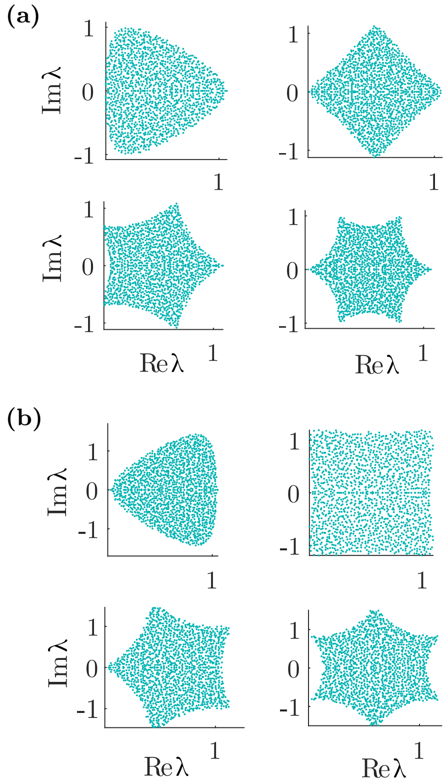

FIG. S6: Eigenvalue distributions for $\alpha = 3$ (top left), $\alpha = 4$ (top right), $\alpha = 5$ (bottom left), and $\alpha = 6$ (bottom right) with (a) $\rho \approx 0.18$, and (b) $\rho \approx -0.18$. Network size is $N = 1600$ and $g_{\rm eff} \approx 1.1$.
